# Vacancies Engineering in Molybdenum Boride MBene Nanosheets to Activate Room‐Temperature Ferromagnetism

**DOI:** 10.1002/adma.202411765

**Published:** 2024-11-02

**Authors:** Liangzhu Zhang, Shucheng Xing, Tian He, Wei‐Bin Wu, An‐lei Zhang, Zhoubin Guo, Pratteek Das, Shuanghao Zheng, Jun‐Yi Ge, Xinliang Feng, Zhimei Sun, Zhong‐Shuai Wu

**Affiliations:** ^1^ State Key Laboratory of Catalysis Dalian Institute of Chemical Physics Chinese Academy of Sciences 457 Zhongshan Road Dalian 116023 China; ^2^ School of Materials Science and Engineering East China University of Science and Technology Shanghai 200237 China; ^3^ Center for Integrated Computational Materials Engineering International Research Institute for Multidisciplinary Science School of Materials Science and Engineering Beihang University Beijing 100191 China; ^4^ Materials Genome Institute Shanghai University Shanghai 200444 China; ^5^ College of Science Nanjing University of Posts and Telecommunications Nanjing 210023 China; ^6^ University of Chinese Academy of Sciences 19 A Yuquan Road, Shijingshan District Beijing 100049 China; ^7^ Center for Advancing Electronics Dresden (cfaed) Faculty of Chemistry and Food Chemistry Technische Universität Dresden 01062 Dresden Germany; ^8^ Max Planck Institute of Microstructure Physics 06120 Halle (Saale) Germany

**Keywords:** MBene, molybdenum boride, nanosheets, room‐temperature ferromagnetism

## Abstract

The rapid development of low energy dissipation spintronic devices has stimulated the search for air‐stable 2D nanomaterials possessing room‐temperature ferromagnetism. Here the experimental realization of 2D Mo_4/3_B_2_ nanosheets is reported with intrinsic room‐temperature ferromagnetic characteristics by vacancy engineering. These nanosheets are synthesized by etching the bulk MAB phase (Mo_2/3_Y_1/3_)_2_AlB_2_ into Mo_4/3_B_2_ nanosheets in ZnCl_2_ molten salt. The Mo_4/3_B_2_ nanosheets show robust intrinsic ferromagnetic properties, with a saturation magnetic moment of 0.044 emu g^−1^ at 300 K, while vacancy‐free MoB MBene exhibits paramagnetism. It is elucidated that the Mo‐vacancy defect generates large density of states near the Fermi surface and spontaneously spin‐split bands through first‐principles calculations, which contributes to the non‐zero magnetic moment in Mo_4/3_B_2_ nanosheets. This work lays the groundwork for activating the magnetic properties of MBene nanosheets by vacancy engineering, offering the possibilities for development of practical spintronic devices.

## Graphical abstract

The experimental realization of 2D Mo‐vacancy molybdenum boride (Mo_4/3_B_2_) MBene nanosheets is demonstrated with intrinsic room‐temperature ferromagnetic character by selectively etching bulk MAB phase (Mo_2/3_Y_1/3_)_2_AlB_2_. The Mo_4/3_B_2_ nanosheets show robust intrinsic ferromagnetic properties with a saturation magnetic moment of 0.044 emu g^−1^ at 300 K.

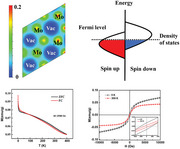

## 1 Introduction

2D materials show rich electronic states and exotic properties that emerge due to the ultrathin thickness, attracting enormous attention in condensed matter physics.^[^ ^1^ ^]^ For a long period, 2D magnetic materials were predicted to be impossible due to the strong intrinsic spin fluctuations in monolayer 2D materials according to Mermin‐Wagner theorem.^[^ ^2^ ^]^ Since then, atomically thin 2D magnetic materials have been realized, including Cr_2_Ge_2_Te_6_, CrI_3_, and Fe_3_GeTe_2_, providing a novel platform for investigating new applications in information storage and magnetic sensing.^[^ ^3^ ^]^ In view of practical applications, the search for 2D materials with intrinsic magnetism and high curie temperature above room temperature is paramount.^[^ ^2^, ^4^ ^]^ Intuitively, examples of recent success, such as MnSe_x_, and VSe_2_, have been achieved.^[^ ^5^ ^]^ However, most of them exhibit poor air stability in the ambient atmosphere, requiring specialized experimental protection (e.g., glove box). Consequently, the development of air‐stable 2D materials with room‐temperature ferromagnetism is of great significance for practical applications.

Recently, a new class of layered transition metal boride named MBene (M = Mo, W, Fe, Cr, Mn) have been reported. The intriguing properties of MBene is the stability in the air due to the robust hybridization of *p*‐orbitals of B atom with *d*‐orbitals of transition metal atom, as experimentally verified in MoB, W_2_B, and CrB Mbenes.^[^ ^6^ ^]^ Moreover, excellent mechanical properties have been reported in MoB_2_ nanosheet with an ultrahigh Young's modulus of 517 GPa.^[^ ^7^ ^]^ The investigation of magnetism in MBene is still very infancy. The theoretical prediction and experimental validation of magnetic property in MnB nanosheet have been realized and it showed a high Curie temperature (*T*_C_) of 585.9 K.^[^ ^8^ ^]^ However, the magnetic properties in other types of MBene without magnetic metal atoms is rarely reported. Therefore, it is an important scientific pursuit to diversify the inventory by introducing magnetic properties to non‐magnetic metal‐based MBene nanosheets for future development of spintronic devices. Defect engineering is regarded as an effective method to introduce magnetism that has been observed in MoS_2_, PtSe_2_, and graphene.^[^ ^9^ ^]^ For instance, layer‐dependent ferromagnetic or antiferromagnetic ground‐state orderings were observed in PtSe_2_ nanosheet by defect engineering. Moreover, exfoliation is a commercial available and scalable production technology which could enable the mass production of magnetic nanosheet powder and ink to be assembled into films by spin coating, vacuum filtration, spray printing, and 3D printing, which would enable the heterostructure and device fabrication for practical application. Therefore, the experimental realization of magnetic properties in MoB MBene by exfoliation through defect engineering represents a meaningful and challenging pursuit.

Here, we adopt a defect engineering strategy to synthesize Mo‐vacancy Mo_4/3_B_2_ MBene nanosheets by using ZnCl_2_ as Lewis acid salt to etch the Al layer and Y atoms from (Mo_2/3_Y_1/3_)_2_AlB_2_ precursor. The etching reaction took place at 700 °C and the reduced zinc particles were subsequently removed in HCl acid. The etched product were intercalated by tetrabutylammonium hydroxide to obtain the Mo_4/3_B_2_ nanosheets with a lateral size of ≈200 nm and thickness of 6.3 nm. The achieved Mo_4/3_B_2_ nanosheets demonstrate two important properties of interest, i.e., excellent stability at ambient temperature without oxidation and intrinsic ferromagnetism with a saturation magnetic moment of 0.04 emu g^−1^ at 300 K. We ascribe the origin of ferromagnetism to Mo‐vacancy defects that generate large density of states near the Fermi surface and spontaneously spin‐split bands, resulting in non‐zero magnetic moment.

## 2 Results and Discussion

**Figure 1:**
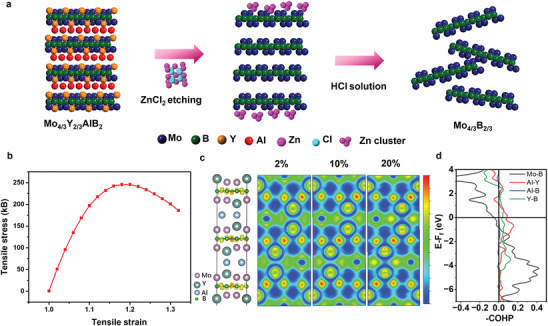
Theoretical analysis of exfoliation possibility of MAB phase into MBene nanosheets. a) Schematic illustration of exfoliation of (Mo_2/3_Y_1/3_)_2_AlB_2_ into Mo_4/3_B_2_ nanosheets by ZnCl_2_ etching. b) The stress‐strain plot of (Mo_2/3_Y_1/3_)_2_AlB_2_ by loading the stress along the Z axis. c) Electron localization function contour plots of (Mo_2/3_Y_1/3_)_2_AlB_2_ along the Z axis by loading the stress from 2 to 20%. d) The ‐COHP curves of (Mo_2/3_Y_1/3_)_2_AlB_2_ under different strains along the Z axis. The loading stress is 20%.

Molten salt etching of (Mo_2/3_Y_1/3_)_2_AlB_2_ MAB into Mo_4/3_B_2_ MBene is schematically illustrated in **Figure** **1**a. The (Mo_2/3_Y_1/3_)_2_AlB_2_ precursor is immersed in ZnCl_2_‐LiCl‐KCl salt and the mixture was heated at 700 °C for 10 h. The molten state Zn^2+^ reacts with the exposed Al atoms and is reduced to Zn metal. The Mo_4/3_B_2_ MBene were formed when excess Zn^2+^ ions reacted with precursor, as described in Equation (1). The Zn particles were dissolved by immersing the prepared powder in dilute HCl acid. Moreover, tetrabutylammonium hydroxide as a frequently used long‐chain alkanes surfactant was used to intercalate and delaminate Mo_4/3_B_2_ MBene to obtain the nanosheets. To understand the reaction mechanism, the Gibbs frees energy was calculated according to Equation (2) which was simplified from Equation (1). The value of Gibbs free energy is ‐236.23 kcal for Equation (2) at 700 °C, suggesting that the reaction is thermodynamically spontaneous (Table S1, Supporting Information). The Mo─B bond remains unchanged with the loading stress increasing from 0 to 20% (Figure 1b,c), while the B─Al bond is weakened, resulting in the relocation of electrons around the Al atom. To better understand the mechanism behind the selective etching of Al and Y atoms from Mo_4/3_Y_2/3_AlB_2_, the crystal orbital Hamilton population (COHP) analysis was conducted. The integrated value area ratio of Mo‐B/Y‐B/Al‐Y is 4.44:1.37:1 under loading strain of 1.2, which indicates that Mo─B bond strength is much stronger than Y‐B and Al‐Y, and the possibility to simultaneously remove the Y and Al atoms during the chemical exfoliation process.

(1) $$\begin{array}{c} 3Mo_{4/3}Y_{2/3}\mathrm{Al}B_{2}+7.5\mathrm{ZnC}l_{2}\to \;3Mo_{4/3}B_{2}+3\mathrm{AlC}l_{3}\\ +\;2\mathrm{YC}l_{3}+7.5\mathrm{Zn} \end{array}$$

(2) $$2Y+3\mathrm{Al}+7.5\mathrm{ZnC}l_{2}\to 3\mathrm{AlC}l_{3}\;+\;2\mathrm{YC}l_{3}\;+\;7.5\mathrm{Zn}$$

**Figure 2:**
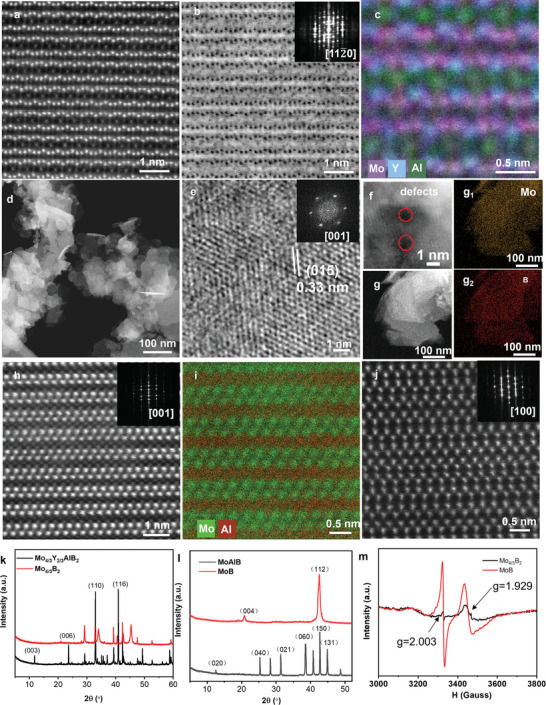
Morphological and structural characterizations of (Mo_2/3_Y_1/3_)_2_AlB_2_ MAB phase and Mo_4/3_B_2_ MBene nanosheets. a) ADF‐STEM image of in‐plane ordered (Mo_2/3_Y_1/3_)_2_AlB_2_ phase along [11$\bar{2}$0] zone axis. b) ABF‐STEM image of (Mo_2/3_Y_1/3_)_2_AlB_2_, inset is the FFT image. c) Atomic EDS mapping of (Mo_2/3_Y_1/3_)_2_AlB_2_. d) Low‐magnification STEM image of exfoliated Mo_4/3_B_2_ MBene nanosheets. e) HRTEM image of Mo_4/3_B_2_ nanosheets along [001] zone axis inset is the FFT image. f) High‐magnification STEM image of Mo_4/3_B_2_ MBene nanosheet. g–g_2_) EDS mapping of Mo_4/3_B_2_ MBene nanosheets. h) ADF‐STEM image of MoAlB phase along [001] zone axis. i) Atomic EDS mapping of MoAlB. j) ADF‐STEM image of MoB MBene. k) XRD patterns of (Mo_2/3_Y_1/3_)_2_AlB_2_ and Mo_4/3_B_2_ MBene. l) XRD patterns of MoAlB and MoB MBene. m) EPR spectra of Mo_4/3_B_2_ MBene and MoB MBene.

The 2D morphology and crystalline structure of (Mo_2/3_Y_1/3_)_2_AlB_2_ were examined by scanning electron microscopy (SEM) and transmission electron microscope (TEM) characterizations. Figure S1 (Supporting Information) shows the layered structure of (Mo_2/3_Y_1/3_)_2_AlB_2_ precursor with a mean lateral size of 5.87 µm. Scanning transmission electron microscopy (STEM) and annular dark field (ADF)‐STEM and annular bright‐field (ABF) STEM images of (Mo_2/3_Y_1/3_)_2_AlB_2_ along [11$\bar{2}$0] zone axis display atomically laminated crystal structure (**Figure** **2**a,b). Sequential layers were observed with the two adjacent layer of Mo and Y and a layer of Al. The brightest atoms are the Mo and Y, while the Al atoms appear dimmer, and B is invisible due to the light atomic mass. The atomic resolution energy dispersive mapping certifies the in‐plane chemical ordering layer of Mo and Y atoms (Figure 2c). STEM image discloses the semi‐transparent layered structure of the exfoliated Mo_4/3_B_2_ MBene nanosheets, with lateral size ranging from 100 to 300 nm (Figures 2d and S2, Supporting Information). Moreover, atomic force microscopy (AFM) image shows flat characteristic with thickness of 6.3 nm for Mo_4/3_B_2_ nanosheet (Figure S3, Supporting Information). High resolution TEM (HRTEM) presents Mo_4/3_B_2_ MBene nanosheets with hexagonal arrangement and lattice distance of 0.32 nm for the (015) crystal plane (Figure 2e). The fast‐Fourier transforms (FFT) manifest as a set of hexagonal spots (Figure 2e), indicating the single crystalline nature and hexagonal symmetry of Mo_4/3_B_2_ nanosheets. The layer distance of 0.43 nm is observed for Mo_4/3_B_2_ nanosheets, which is much smaller than that of (Mo_2/3_Y_1/3_)_2_AlB_2_ layer distance (0.76 nm), certifying the removal of Al layer (Figure S4, Supporting Information). HADDF‐STEM image of layered structure Mo_4/3_B_2_ nanosheet did not show any adjacent atoms near Mo layers, suggesting that ‐Cl group does not covalently bond with the nanosheets (Figure S4, Supporting Information). The uniform dispersion of Mo and B element in EDS mapping confirms the chemical composition of Mo_4/3_B_2_ nanosheets (Figure 2g–g_2_). Figure 2f shows high resolution ADF‐STEM of Mo_4/3_B_2_ nanosheet and two sub‐nanometer etched cavity are observed, suggesting the Mo‐vacancy defects on nanosheet. Further, the pristine MoB MBene nanosheets without Mo vacancies were synthesized through molten salt etching by using micrometer size MoAlB precursor (Figure S5a,b, Supporting Information). The as‐exfoliated MoB MBene exhibits an accordion‐like morphology (Figure S5c, Supporting Information). ADF‐STEM unravels that the structure of MoAlB phase is formed by two layers of Mo atoms sandwiched by two layers of Al atoms along the [001] plane, which was further observed in the atomic resolution EDS mapping picture (Figure 2h,i). After extracting the Al layers by molten salt etching in ZnCl_2_, the MoB MBene is obtained. Figure 2j confirms that the MoB MBene crystal structure is *α*‐MoB with stacking fault along the [001] zone axis.^[^ ^10^ ^]^

We characterized the X‐ray diffraction (XRD) patterns of (Mo_2/3_Y_1/3_)_2_AlB_2_ and Mo_4/3_B_2_ nanosheets as shown in Figure 2k. Compared with the bulk phase (Mo_2/3_Y_1/3_)_2_AlB_2_, the (003) and (006) peaks were almost diminished, indicating the successful etching of Al from (Mo_2/3_Y_1/3_)_2_AlB_2_ into layered Mo_4/3_B_2_ nanosheets.^[^ ^11^ ^]^ The disappearance of the (003) and (006) reflection in MoAlB pattern after etching, compared to that of the synthesized MoB MBene, verifies the complete removal of Al layers.^[^ ^12^ ^]^ The inductively coupled plasma optical emission spectrometry further shows only 0.66 wt% Y element remained in the Mo_4/3_B_2_ MBene (Table S2, Supporting Information), validating the removal of Y in (Mo_2/3_Y_1/3_)_2_AlB_2_, which is the origin of Mo‐vacancy defect. X‐ray photoelectron spectroscopy of Y3d shows the diminishing of the fitted peak of Mo_4/3_B_2_ nanosheets in comparison with that of (Mo_2/3_Y_1/3_)_2_AlB_2_ (Figure S6a, Supporting Information), further proving the selective etching of Y element. Two significant electron spin resonance signals at *g* =  2.003 and 1.929 were observed for Mo_4/3_B_2_ MBene compared with MoB MBene in Figure 2m, which can be ascribed to the large increments of the paramagnetic center of Mo^5+^ (Figure S6b, Supporting Information), which could be assigned to the appearance of Mo vacancies.^[^ ^13^ ^]^

**Figure 3:**
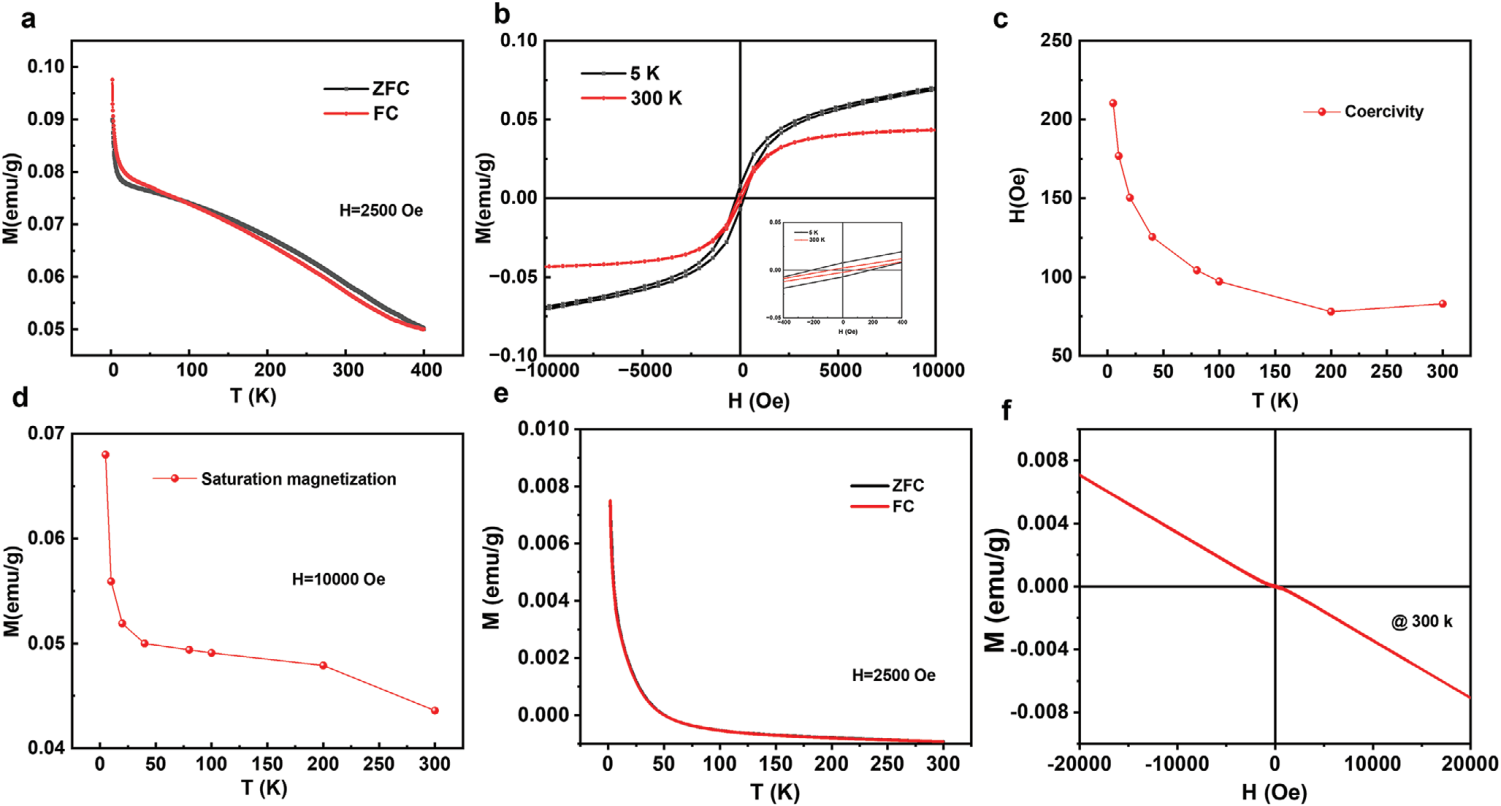
Magnetization characterization of Mo_4/3_B_2_ and MoB MBene. a) Temperature dependent ZFC, and FC magnetization curve measured under external field of 2500 Oe of Mo_4/3_B_2_ nanosheets. b) Magnetization loops of Mo_4/3_B_2_ nanosheets at 5 and 300 K. The inset shows the enlarged view of the hysteresis loop. c) Coercivity at different temperatures of Mo_4/3_B_2_ nanosheets. d) Saturation magnetization versus temperature curve of Mo_4/3_B_2_ nanosheets under external field of 10 000 Oe. e) Temperature dependent ZFC, and FC magnetization curve measured under external field of 2500 Oe of MoB nanosheets. f) Magnetization loops of MoB MBene at 300 K.

The magnetic properties of Mo_4/3_B_2_ nanosheets were studied by magnetization curves versus temperature (M‐T) under both zero field‐cooling (ZFC) and field‐cooling (FC) modes together with the isothermal magnetization loops (M‐H) using magnetic properties measurement system (**Figure** **3**). From Figure 3a, the M‐T curve of Mo_4/3_B_2_ nanosheets does not follow the typical Curie‐Weiss behavior. For most paramagnetic materials, the M‐T curve is saturated at high temperatures. However, in Mo_4/3_B_2_ nanosheets, the magnetization still decreases rapidly with increasing temperature up to room temperature, which indicates that Mo_4/3_B_2_ exhibits ferromagnetism at room temperature. Instead, the magnetization keeps going down with an increasing trend close to the room temperature and unsaturated till 1000 °C (Figure S7, Supporting Information), suggesting a high *T*_C_ above 300 K. Furthermore, well‐defined hysteresis loops from 5 to 300 K verify the room‐temperature ferromagnetic ordering for Mo_4/3_B_2_ nanosheets (Figure 3b). As shown in Figure 3c,d, coercivity and saturation magnetization at different temperatures are extracted from the M‐H curves, where both decrease with increasing temperature. Coercivity of ≈210.4 and 83 Oe and saturation magnetization of ≈0.068 and 0.044 emu g^−1^ are observed at 5 and 300 K M‐H curves, respectively, which suggests the soft ferromagnetic property. It should be noted that saturation magnetization of 0.044 emu g^−1^ for Mo_4/3_B_2_ nanosheets at 300 K is much higher than the values reported in Ti_3_C_2_ (0.002 emu g^−1^) and Nb_2_C (0.013 emu g^−1^) MXene.^[^ ^14^ ^]^ There are no ferromagnetic impurities in Mo_4/3_B_2_ MBene and the copper sample pole exhibits diamagnetic properties. Thus, it is clear that the ferromagnetism observed is inherent in our samples. However, compared with Mo_4/3_B_2_ nanosheets, the M‐T curve of MoB MBene exhibits a typical paramagnetic behavior, which is well explained by the Curie‐Weiss law (Figures 3e and S8, Supporting Information). It should be noted that the negative signals of the M‐T curve could be induced by the copper sample pole during the test. Although the background signal appears here, it does not affect the magnetic behavior of the sample, and we have mainly characterized the weak paramagnetism of MoB MBene. The fitting results show that the Curie constant is 2.83 × 10^−5^ emu K g^−1^ Oe^−1^ and Curie‐Weiss temperature is −6.90 K. The negative Curie‐Weiss temperatures suggest that the MoB MBene exhibits paramagnetism with a weak antiferromagnetic ordering. In addition, the M‐H curve at 300 K discloses that weak paramagnetism is covered by the antiferromagnetic signal of copper sample pole (Figure 3f). This is a strong evidence that the MoB MBene devoid of vacancies exhibits extremely tiny paramagnetism. This comparison with MoB MBene suggests that vacancy engineering can successfully make Mo_4/3_B_2_ MBene show robust room‐temperature ferromagnetism.

**Figure 4:**
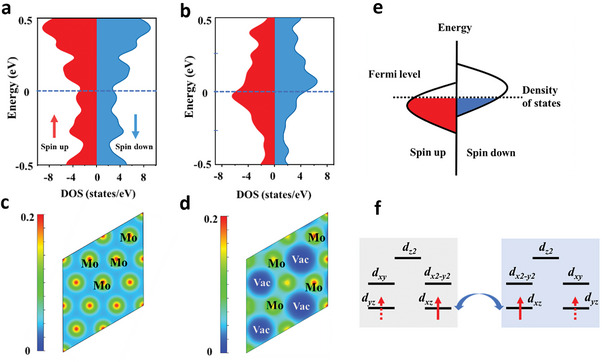
The first‐principles calculations of the Mo‐vacancy defect induced room‐temperature ferromagnetism in 2D Mo_4/3_B_2_. a) Density of states of MoB and b) Mo_4/3_B_2_ MBene. c) Charge density of MoB and d) Mo_4/3_B_2_. e) Schematic band structure for the Stoner model of ferromagnetism. f) Magnetic coupling diagram of 2D Mo_4/3_B_2._

To bolster our understanding of the Mo‐vacancy defect‐induced magnetic moments in 2D Mo_4/3_B_2_, we conducted comprehensive first‐principles calculations. Our results, presented in **Figure** **4**a–d, unequivocally show that the electrons in Mo_4/3_B_2_ exhibit notably more itinerant characteristics compared to MoB, as evidenced by the density of states and charge density analyses. This distinction is further emphasized by the significant contribution of the Mo‐vacancy defect, strategically positioned near the surface, which gives rise to a pronounced density of states near the Fermi surface in the Mo_4/3_B_2_ nanosheet. Importantly, we find that this enhancement in the density of states, coupled with the Coulomb repulsion strength U, satisfies the crucial Stoner criterion Ug(*E*_F_) ≥ 1 for itinerant ferromagnetism, where g(*E*_F_) represents the density of states at the Fermi level.^[^ ^15^ ^]^ Consequently, this condition predicts the possibility of spontaneously spin‐split bands, ultimately leading to a non‐zero magnetic moment and spin polarization of the system (Figure 4e), in accordance with the Stoner criterion.^[^ ^16^ ^]^

To delve deeper into the intricate mechanisms underpinning the magnetic exchange facilitated by the Mo vacancy defect in Mo_4/3_B_2_, we deliberately consider the magnetic exchange constant *J* and orbital coupling. Specifically, we observe that the *d* orbitals of Mo_4/3_B_2_ are split into distinct states due to the perturbation induced by the trigonal crystal field, comprising a solitary *d_z2_* orbital and two doubly degenerate pairs, namely (*d_xy_ +d_x2‐y2_*) and (*d_xz_+d_yz_*) (Figure S9, Supporting Information). Based on the rigorous Bader charge analysis, we ascertain that each Mo atom transfers a single electron to a B atom, resulting in a distinct spin occupation pattern of Mo, as depicted in Figure 4f. Notably, the magnetic exchange couplings are predominantly mediated by the spin‐spin interactions between *d_xz_‐d_xz_* and *d_yz_‐d_yz_* orbitals, which aligns seamlessly with our calculated orbital‐projected magnetic exchange constant *J* presented in Figure S10 (Supporting Information). These findings provide a compelling theoretical framework to substantiate our claims of intrinsic ferromagnetism in Mo_4/3_B_2_, rendering our results both rigorous and convincing.

In summary, as the research of magnetism in MBene system is in the initial stage, this strategy of defects engineering to generate the room‐temperature magnetism is demonstrated in Mo‐vacancy Mo_4/3_B_2_ nanosheets. Expanding magnetic properties of molybdenum boride MBene would open the door for synthesizing a broad range of molybdenum boride 2D room temperature magnets. The room temperature Mo_4/3_B_2_ magnets also provide a large room for designing promising devices in different fields, such as spintronics and magnetically separable nanocarriers. Therefore, our work provides a new avenue for developing magnetic nanomaterials from emerging MBene nanosheets to accelerate the application of practical spintronic devices.

## 3 Experimental Section

### Materials

Molybdenum (Mo, metal basis, 99.9%, Tianjin Special Powder Research Institute), yttrium (Y, metal basis, 99.9%, Tianjin Special Powder Research Institute), aluminum (Al, metal basis, 99.9%, Tianjin Special Powder Research Institute), boron (B, metal basis, 99.9%, Tianjin Special Powder Research Institute), zinc chloride (ZnCl_2_, Shanghai Aladdin Biochemical Technology co., Ltd.), molybdenum aluminum boride (MoAlB, Jilin 11 Technology Co., Ltd), lithium chloride (LiCl, Shanghai Aladdin Biochemical Technology co., Ltd.), potassium chloride (KCl, Shanghai Aladdin Biochemical Technology co., Ltd.), and hydrochloric acid (HCl, Shanghai Aladdin Biochemical Technology co., Ltd.) were used without further treatment.

### Preparation of Mo_4/3_B_2_ Nanosheets

The MAB (Mo_2/3_Y_1/3_)_2_AlB_2_ precursor was synthesized by solid‐state reaction. In details, 5.11 g Mo, 2.37 g Y, 1.07 g Al, and 0.43 g B powder were homogenously grinded in an agate mortar and loaded in alumina boat. The mixture was sintered at chemical vapor deposition furnace under the protection of Ar gas. The furnace was first ramped up to 1400 °C in 280 min from room temperature, kept at 1400 °C for 480 min, and cooled to room temperature in 280 min. Mo_4/3_B_2_ nanosheets was prepared by Lewis acid molten salt etching. In brief, 2.2 g (Mo_2/3_Y_1/3_)_2_AlB_2_, 13.6 g ZnCl_2_, 7.4 g KCl, and 4.2 g LiCl were grinded and loaded in alumina boat. The mixture was sintered at chemical vapor deposition furnace under the protection of Ar gas. The furnace was first ramp up to 700 °C in 140 min from room temperature, kept at 700 °C for 600 min, and cooled to room temperature in 140 min. The obtained mixture was washed by DI water to dissolve the remaining ZnCl_2_, LiCl, and KCl for three times. The remaining products were collected by vacuum filtration on a nylon membrane. The product was added into a 100 mL HCl acid solution (1 mol L^−1^) and stirred for 4 h to remove the Zn particles on Mo_4/3_B_2_ MBene. Moreover, 20 mL tetrabutylammonium hydroxide (25 wt%) solution was used to intercalate the Mo_4/3_B_2_ MBene by magnetic stirring for 10 h. After washing the intercalated Mo_4/3_B_2_ MBene with DI water for four times, Mo_4/3_B_2_ nanosheets powder was collected by centrifugating at 6000 rpm for 5 min and finally freeze dried.

### Preparation of MoB MBene

The MoB MBene was prepared by using the Lewis acid molten salt etching, which was similar to the preparation of Mo_4/3_B_2_ nanosheets. 1.3 g MoAlB, 13.6 g ZnCl_2_, 7.4 g KCl, and 4.2 g LiCl were grinded and loaded in an alumina boat. The mixture was sintered in a chemical vapor deposition furnace under the protection of Ar gas. The furnace was first ramped up to 700 °C in 140 min from room temperature, kept at 700 °C for 600 min, and cooled to room temperature in 140 min. The obtained mixture was washed by DI water to dissolve the remaining ZnCl_2_, LiCl, and KCl for three times. The remaining products were collected by vacuum filtration on a nylon membrane. The product was added into a 100 mL HCl acid solution (1 mol L^−1^) and stirred for 4 h to remove the Zn particles on MoB MBene. MoB MBene powder was collected by centrifugation at 6000 rpm for 5 min and thus freeze dried.

### Materials Characterization

The morphology, crystal structure, and thickness of (Mo_2/3_Y_1/3_)_2_AlB_2_, Mo_4/3_B_2_ MBene nanosheets, MoYAlB, and MoB MBene were characterized by the scanning electronic microscopy (SEM, JSM‐7900F, JEOL), aberration‐corrected transmission electron microscopy (TEM, JEM‐ARM 300F GRAND ARM, JEOL), atomic force microscopy (AFM, Multi‐Mode 3D, Veeco), optical microscope (Keyence), X‐ray diffraction (XRD, SmartLab, Rigaku), X‐ray photoelectron spectra (XPS, Escalab 250 Xi+, Thermofisher), and Raman spectroscopy (LabRAM HR800, HORIBA JOBIN YVON corporation). Elemental analysis of Mo, Y, Al, and B in Mo_4/3_B_2_ nanosheets was detected by inductively coupled plasma optical emission spectrometry (ICP‐OES, Agilent 720es). FEI Scios Dual‐Beam Focused Ion Beam (FIB) equipped with a SEM was used to form the cross sections and to take the cross sectional images. The electron and ion beams intersect at an angle of 52°and at a coincident point near the sample surface, allowing immediate SEM imaging of the FIB‐milled surface. The milling was performed with a 15 nA, 30 kV Gallium beam, followed by cleaning steps at 5 nA and then 0.5 nA. Magnetic property of Mo_4/3_B_2_ nanosheets and MoB MBene were tested on a superconducting quantum interference device (MPMS‐3, Quantum Design Inc.). Temperature‐dependent magnetization for Mo_4/3_B_2_ MBene between 300–1000 K was measured by the vibrating sample magnetometer (VSM) option of the physical property measurement system (PPMS, Quantum Design Inc.).

### Theoretical Calculations

The calculations were performed in the framework of density functional theory as implemented in the Vienna ab initio simulation package (VASP). The projector augmented wave (PAW) potential was used with the plane‐wave cutoff energy set as 500 eV, and the Brillouin zone is integrated with 7 × 7 × 1 *k*‐point mesh. A vacuum region more than 20 Å was modeled to avoid interaction between neighboring slabs.

The exchange constants were calculated using the Green's function method by the TB2J code interfaced with siesta. The magnetic exchange coupling constants were derived by comparing the energy change due to a small spin rotation between the Heisenberg model and Green's‐function energy expression, utilizing the local force theorem. The Heisenberg exchange coupling constant *J* for local spin vectors of unit length can be obtained as the imaginary part of an integral over the trace of products of the Green's functions for *α* and *β* spins and so‐called on‐site potentials *V̂_A_* and *V̂_B_* of the two magnetic sites:

(3) JGreenunitF=−14πIm∫−∞εFdεTrV^AG^αV^BG^β

## Conflict of Interest

The authors declare no conflict of interest.

## Supporting information



Supporting Information

## Data Availability

The data that support the findings of this study are available from the corresponding author upon reasonable request.
