# Supplementary material for: Vacancies Engineering in Molybdenum Boride MBene Nanosheets to Activate Room‐Temperature Ferromagnetism
Source: Adv Mater. 2024 Nov 2;37(1):2411765. doi: 10.1002/adma.202411765 (PMC11707573; doi:10.1002/adma.202411765)
Supplement: Supplementary file 1 — Supporting Information [file ADMA-37-2411765-s001.pdf]

ADVANCED MATERIALS

Supporting Information

for *Adv. Mater.*, DOI 10.1002/adma.202411765

Vacancies Engineering in Molybdenum Boride MBene Nanosheets to Activate Room-Temperature Ferromagnetism

Liangzhu Zhang, Shucheng Xing, Tian He, Wei-Bin Wu, An-lei Zhang, Zhoubin Guo, Pratteek Das, Shuanghao Zheng, Jun-Yi Ge*, Xinliang Feng*, Zhimei Sun* and Zhong-Shuai Wu**

Supporting Information

Vacancies Engineering in Molybdenum Boride MBene Nanosheets to Activate Room-temperature Ferromagnetism

Liangzhu Zhang[*], *Shucheng Xing, Tian He, Wei-Bin Wu, An-lei Zhang, Zhoubin Guo, Pratteek Das, Shuanghao Zheng, Jun-Yi Ge*[*], *Xinliang Feng*[*], *Zhimei Sun*[*], *Zhong-Shuai Wu*[*]

Table S1|Gibbs Free Energy ΔGr of the reaction of $ZnCl_2$ with Al-site elements in $(Mo_{2/3}Y_{1/3})_2AlB_2$ at 700 °C.

**T**	**ΔH**	**ΔS**	**ΔG**
**°C**	**kcal**	**cal/K**	**kcal**
0.000	-237.116	-21.875	-231.141
100.000	-237.335	-22.587	-228.907
200.000	-211.770	32.317	-227.061
300.000	-210.223	35.289	-230.449
400.000	-229.889	2.565	-231.615
500.000	-218.655	18.889	-233.259
600.000	-220.611	16.512	-235.029
700.000	-230.334	6.067	-236.238
800.000	-216.096	20.223	-237.799
900.000	-216.735	19.655	-239.792
1000.000	-217.409	19.103	-241.730

The reaction can be briefed as $2Y+3Al+7.5ZnCl_2 \rightarrow 3AlCl_3+2YCl_3+7.5Zn$. ΔH (f stands for formation) and ΔS can be obtained from the HSC software. ΔGr is calculated from the equation of $\Delta Gr=\Delta H-T\Delta S$.

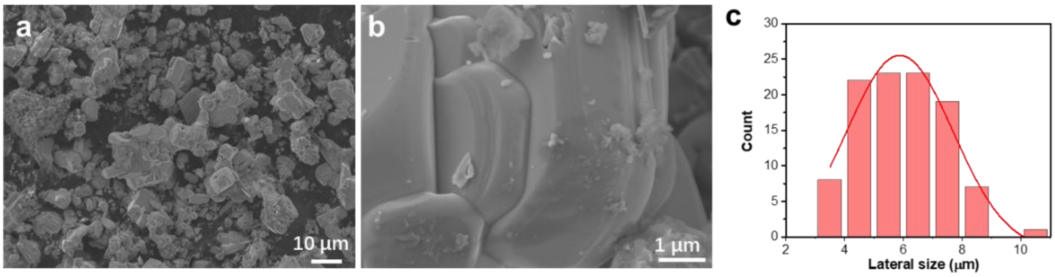

Figure S1.Morphology characterization of $(Mo_{2/3}Y_{1/3})_2AlB_2$ phase. a, SEM image of $(Mo_{2/3}Y_{1/3})_2AlB_2$. b, High-magnification SEM image of $(Mo_{2/3}Y_{1/3})_2AlB_2$. c, Lateral size distribution of $(Mo_{2/3}Y_{1/3})_2AlB_2$ particles counted from 103 individual particles and a Gaussian fit (red). The average lateral size is 5.87 µm and the standard deviation is 0.16 µm.

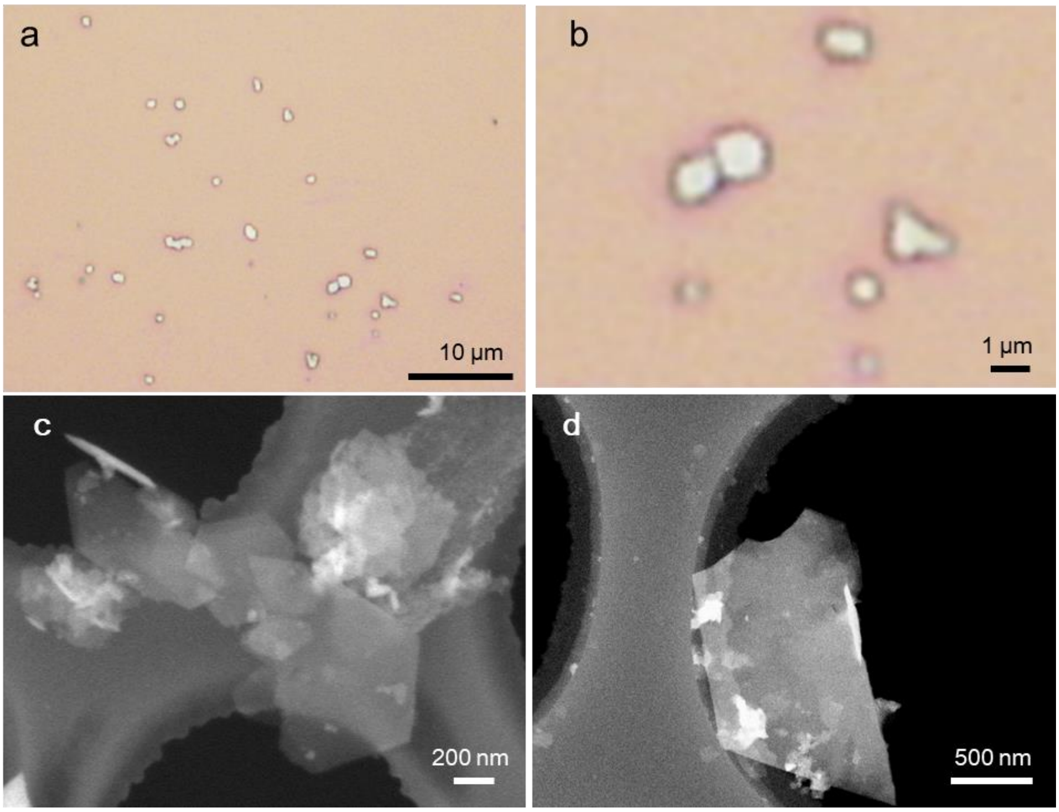

Figure S2. Morphology characterization of $Mo_{4/3}B_2$ nanosheets. a, Optical image of a $Mo_{4/3}B_2$ nanosheets with sub-micrometer size deposited on 300 nm SiO_2/Si. b, Enlarged view of (a). (c) and (d) ADF-STEM image of $Mo_{4/3}B_2$ nanosheets.

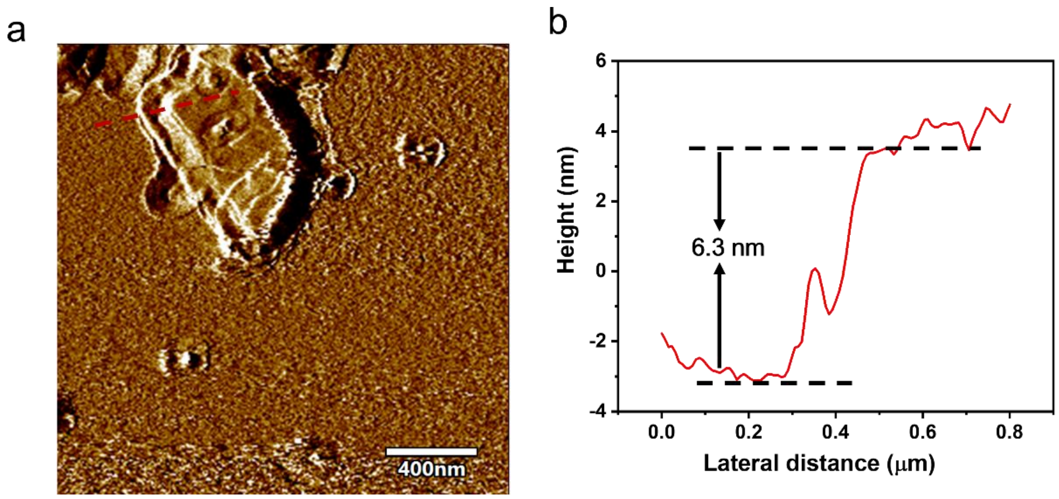

Figure S3. a, AFM image of $Mo_{4/3}B_2$ nanosheet. **b**, height profile of marked line in (a) , showing a thickness of 6.3 nm.

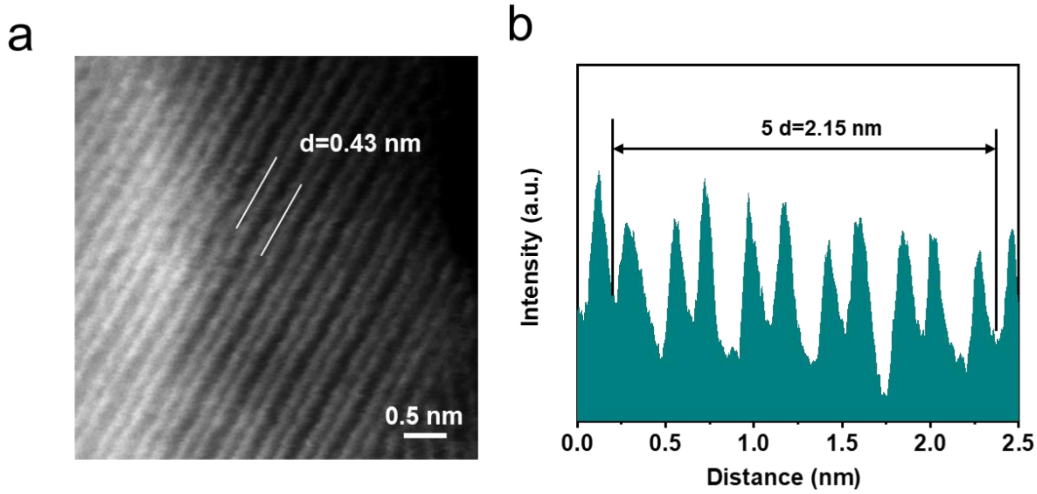

Figure S4. a, ADF-STEM image of the layered structure of $Mo_{4/3}B_2$ nanosheets. b, layer distance height profiles of $Mo_{4/3}B_2$ MBene nanosheets.

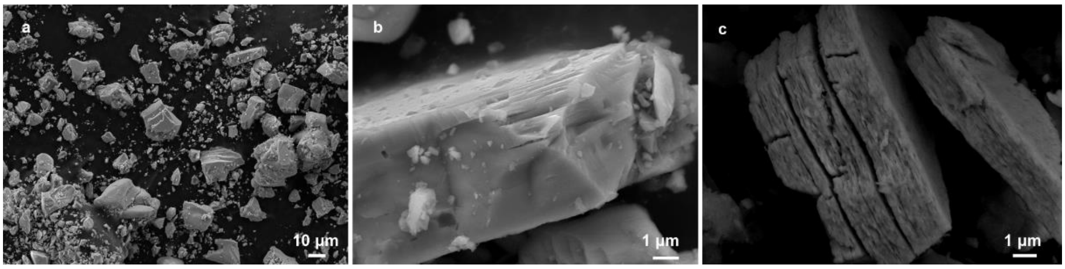

Figure S5. Morphology characterization of MoAlB and MoB MBene. a, SEM image of MoAlB. b, High-magnification SEM image of MoAlB. c, SEM image of MoB MBene.

Table S2. Element contents of $Mo_{4/3}B_2$ MBene, MoAlB phase, and MoB MBene tested by ICP.

Sample	Measured by ICP (wt. %)			
	Mo	Y	Al	B
$Mo_{4/3}B_2$	61.99	0.66	5.38	8.56
MoAlB	65.33		22.07	6.98
MoB	75.3		7.68	7.34

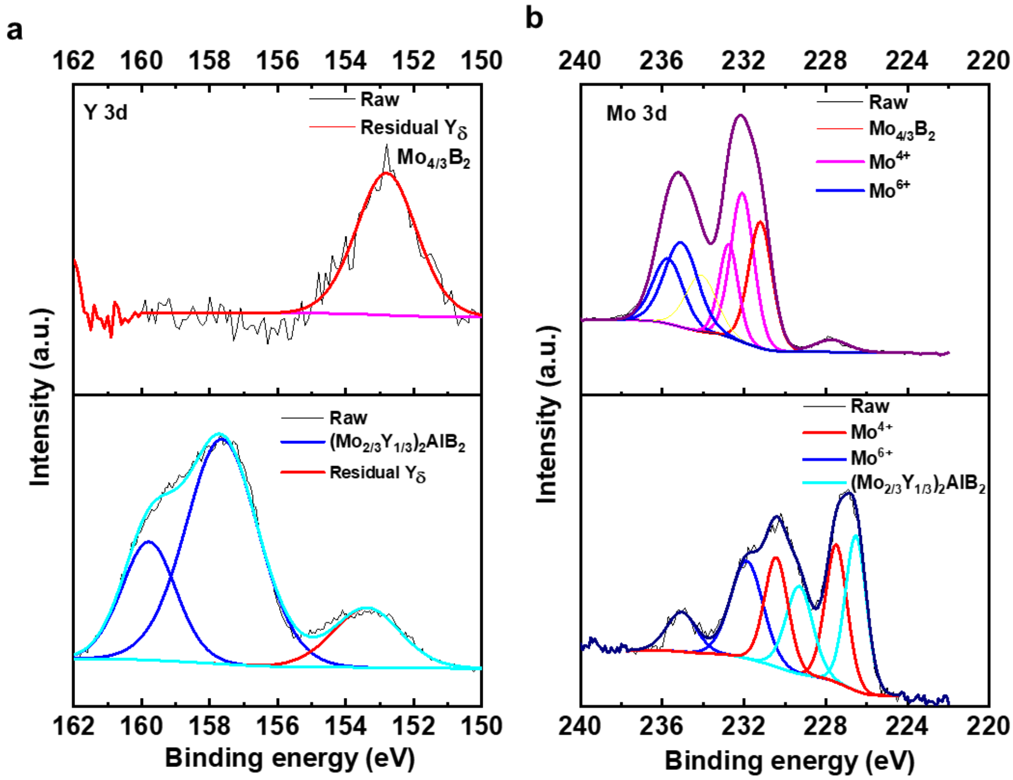

Figure S6. a, XPS high-resolution spectra of $(Mo_{2/3}Y_{1/3})_2AlB_2$ bulk powder and $Mo_{4/3}B_2$ MBene with peak fitting of Y 3d, (b) Mo 1s. δ is residual Y, which closes to noise level for the $Mo_{4/3}B_2$ MBene. In the fitted pattern of Mo 3d peaks, it shows that the content of Mo^{5+} is much increased in $Mo_{4/3}B_2$ compared to that of $(Mo_{2/3}Y_{1/3})_2AlB_2$.

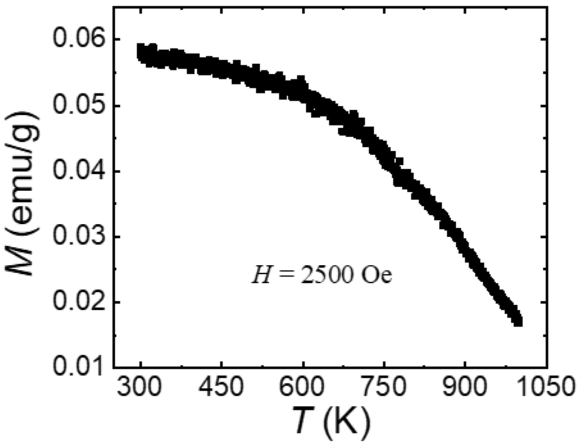

Figure S7. Temperature dependent magnetization for $Mo_{4/3}B_2$ nanosheets between 300 K - 1000 K via vibrating sample magnetometer of PPMS.

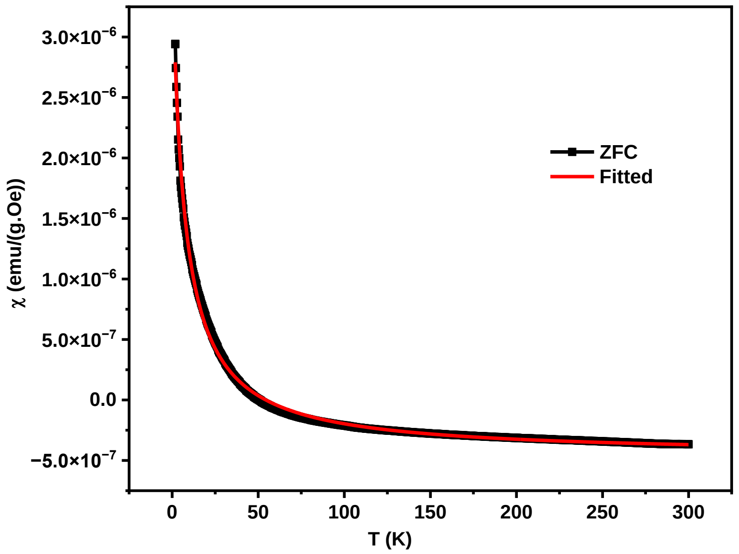

Figure S8. The temperature-dependent susceptibility (χ–T) curves of MoB MBene under 2500 Oe. The black dot line is experimental data under magnific filed of 2500 Oe. The red line is fitted to Curie–Weiss law.

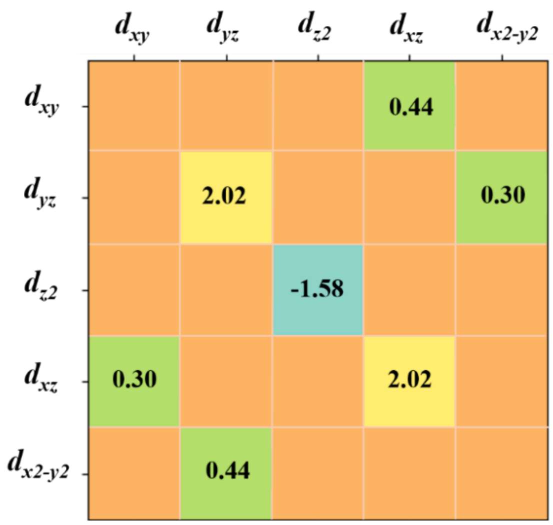

Figure S9. Orbital-resolved magnetic exchange constants between the nearest Mo atoms. The orange lattices denote the value of zero. The positive (negative) values indicate the ferromagnetic (antiferromagnetic) interactions.

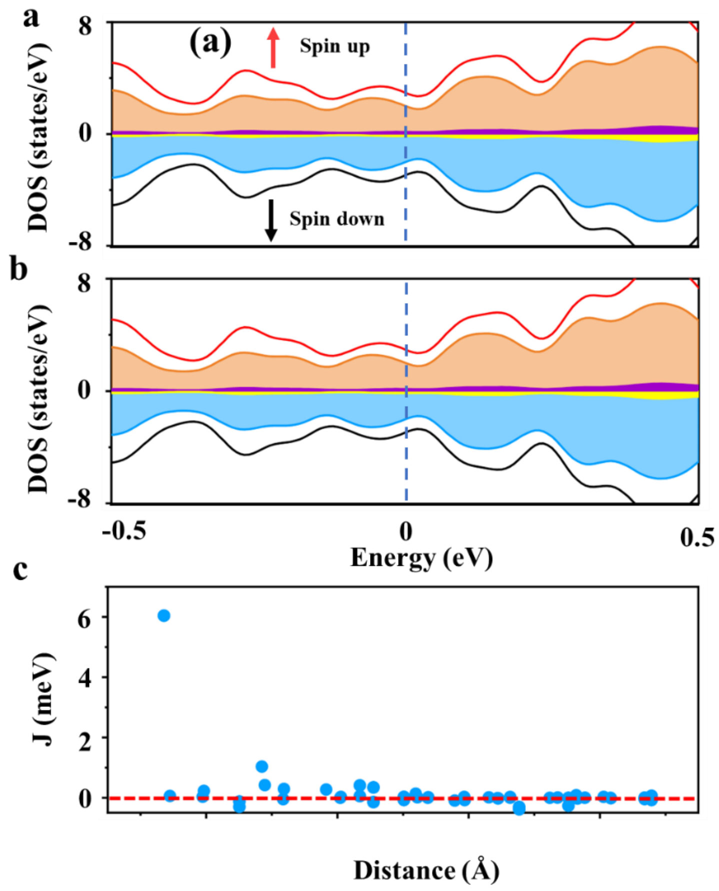

Figure S10. a, Orbital–resolved density of states of 2D MoB and (b) $Mo_{4/3}B_2$ MBene. The red and black lines denote the spin-up and spin-down states of all electrons. The orange and blue shadows denote the spin-up and spin-down states of *d* orbitals of Mo atoms. The purple and yellow shadows denote the spin-up and spin-down states of B atoms. c, Distance-dependent magnetic exchange interactive constants.
